# Higher vs. lower fluid volume for septic shock: clinical characteristics and outcome in unselected patients in a prospective, multicenter cohort

**DOI:** 10.1186/cc11333

**Published:** 2012-05-08

**Authors:** Søren H Smith, Anders Perner

**Affiliations:** 1 Department of Intensive Care, Copenhagen University Hospital, Rigshospitalet, Blegdamsvej 9, DK-2100 Copenhagen, Denmark

## Abstract

**Introduction:**

Patients with septic shock require fluid, but the optimum amount is unknown. Therefore we assessed patient characteristics and outcome associated with fluid volume in unselected patients with septic shock including those with three days of shock.

**Methods:**

We conducted a prospective, multicenter, observational study of all adult patients with septic shock during a 3-month study period at six general ICUs: three in university hospitals and three in regional hospitals. After day 1 and 3 of shock, patients were divided into two groups according to the overall median fluid volumes. Characteristics between these groups were compared using non-parametric and Chi-square statistics.

**Results:**

The 164 included patients received median 4.0 l (IQR 2.3-6.3) of fluid during the first day of septic shock. Patients receiving higher volumes (> 4.0 l) on day 1 had higher p-lactate (3.4 (2.2-5.5) vs. 2.0 (1.6-3.0) mmol l^-1^, *P *< 0.0001) compared to those receiving lower volumes. In contrast simplified acute physiology score (SAPS) II (54 (45-64) vs. 54 (45-67), *P* = 0.73), sequential organ failure assessment (SOFA) score (11 (9-13) vs. 11 (9-13), *P *= 0.78) and 90-day mortality (48 vs. 53%, *P *= 0.27) did not differ between groups. The 95 patients who still had shock on day 3 had received 7.5 l (4.3 - 10.8) of fluid by the end of day 3. Patients receiving higher volumes (> 7.5 l) had higher p-lactate (2.6 (1.7-3.4) vs. 1.9 (1.6-2.4) mmol l^-1^, *P *< 0.01) on day 3 and lower 90-day mortality (40 vs. 62%, *P *= 0.03) than those receiving lower volumes in spite of comparable admission SAPS II (53 (46-67) vs. 55 (49-62), *P *= 0.47) and SOFA scores on day 3 (10 (8-13) vs. 11 (10-14), *P *= 0.33).

**Conclusions:**

In this cohort of unselected ICU patients with septic shock, initial fluid volume was not associated with mortality. In patients with shock for three days or more, higher fluid volumes including crystalloids, colloids and blood products were associated with reduced mortality.

## Introduction

Sepsis is characterized by inflammation-induced endothelial dysfunction leading to vascular leakage and vasodilatation. Ultimately, this results in relative and absolute hypovolemia, organ hypoperfusion, and septic shock. If shock persists, the result is progressive multiple organ failure and high mortality [1].

The circulatory treatment of patients with septic shock is to administer intravenous (IV) fluids and inotrope/vasopressor drugs to optimize cardiac preload and organ perfusion [2]. It is currently unknown whether a strategy using higher or lower fluid volume is better. The Surviving Sepsis Campaign (SSC) recommends goal-directed optimization in the first 6 hours followed by fluid challenges in case of persistent hypoperfusion. The former is based on one relatively small, single-center, randomized clinical trial (RCT) [3] and the latter on expert opinion. Even though these approaches may be physiologically rational, the recommendations illustrate the low level of evidence for fluid volume in septic shock.

Recent cohort studies [4-6] indicated that positive fluid balance was associated with increased mortality. However, neither of these studies was done in unselected patients with septic shock, who may have the highest benefit of fluid therapy. If fluids benefit these patients, this may be better captured by volumes of IV fluid given instead of by fluid balance, which is affected by fluid output.

Therefore, our aim was to describe patient characteristics and mortality in a cohort of unselected patients with septic shock treated with higher versus lower volumes of IV fluids during shock. As patients with persisting septic shock may present a subgroup needing higher volumes of fluid, we performed subgroup analyses of those patients remaining in shock for 3 days or more.

## Materials and methods

This was a prospective, cohort study of consecutive patients with septic shock in the intensive care unit (ICU). Patients were included from the general ICUs at Rigshospitalet and Herlev, Glostrup, Gentofte, Køge, and Hillerød hospitals (Denmark) during a 3-month study period. Three of these are university hospitals and three are regional hospitals. The 3-month study period varied between units, but all were within the period of 1 February to 30 June 2009. The staff at the units was unaware of the study.

All patients with septic shock in the study period were included. Septic shock was defined as (a) documented or suspected infection; (b) two of the following: temperature of less than 36°C or more than 38°C, leukocyte count of less than 4 × 10^9^/L or more than 12 × 10^9^/L, respiratory frequency of more than 20 breaths per minute or mechanical ventilation, or heart rate of more than 90 beats per minute; (c) signs of organ failure - sequential organ failure assessment (SOFA) scores of 2 or more in the cerebral, kidney, liver, lung, or coagulation component - or plasma lactate of more than 2 mmol/L; and (d) need of vasopressor infusion to maintain systolic blood pressure of more than 90 mm Hg after initial fluid resuscitation [7]. The Ethics Committee of the Capital Region and the Danish Data Protection Agency and the National Board of Health approved the study. Consent was waived because all interventions and measurements were clinically indicated.

### Data acquisition

Data were registered from the time septic shock was diagnosed in the ICU and continued as long as the patient received vasopressor or inotropic therapy, so that any day with one of these therapies represented a shock day. For patients who had shock on ICU admittance, data from the pre-ICU period were not registered, as we previously found low quality of such data in a comparable cohort [8].

The following general characteristics were recorded: gender, age, if emergency surgery had been performed, site of infection, simplified acute physiology score II (SAPS II) based on observations during the first 24 hours in the ICU, and SOFA scores on days of shock. SAPS II and SOFA scores of patients who stayed in the ICU less than 24 hours were determined on the basis of the data available from the time they did stay in the ICU. The mortality 90 days after inclusion was registered from the national Danish hospital administration system (GS Open) by using national identification numbers. This ensured full mortality follow-up of Danish citizens. Two patients were non-Danish citizens and were alive at hospital discharge and therefore counted as survivors in the 90-day mortality analysis.

The following interventions were recorded every day during shock: the type and volume of administered fluid, including crystalloids (isotonic NaCl, Ringer's lactate or acetate, other electrolyte solutions, or isotonic glucose); colloids (albumin, hydroxyethyl starch (HES), or dextran; gelatin was not used in the present cohort); blood products (red blood cells (RBCs), fresh frozen plasma, or platelet concentrate; blood products were included because data indicate that these are used for resuscitation in Scandinavian ICUs [9,10]); nutrition given (any nutritional product containing glucose of greater than 5%, amino acids, or lipids); use of corticosteroids, invasive ventilation, or renal replacement therapy; and type and maximum dose of infused inotrope/vasopressor drugs. Dopamine doses were converted into comparable doses of norepinephrine so that 1 μg/kg per minute dopamine equaled 0.01 μg/kg per minute norepinephrine [11]. The converted dose of dopamine was added to any norepinephrine dose to give the maximum vasopressor dose. None of the patients received vasopressin.

The following monitoring characteristics were recorded every day during shock: highest values of plasma lactate and lowest central venous oxygen saturation (ScvO_2_) in the superior caval vein and any use of any device for cardiac output measurement. All data were collected on paper case report forms by a single investigator (SHS) and entered into an Excel (Microsoft Corporation, Redmond, WA, USA) data sheet.

### Statistics

Patients were divided into two groups according to the full cohort's median IV fluid volume administered in the first 24 hours of shock. Volumes of fluid given as nutrition were not included, because the purpose of the study was to assess the effects of IV fluid treatment. Patients who still had shock on day 3 after inclusion were divided into two groups according to the median fluid volume administered in the first 3 days of shock. This way of comparing higher versus lower fluid volume was previously done for the first 6 and 24 hours in patients with severe sepsis and septic shock, respectively [8,12]. The 3-day time point was chosen because it was used in the SOAP (Sepsis Occurrence in Acutely Ill Patients) cohort [4].

Data were expressed as median and interquartile range (IQR) for continuous variables and as number of patients and percentage for categorical variables. Mann-Whitney *U *test was used to compare unpaired continuous data because the fluid data were not normally distributed, and chi-squared test was used for categorical data. *P *values of less than 0.05 were used as the level of statistical significance, and GraphPad Prism version 5.04 (GraphPad Software, La Jolla, CA, USA) was used to analyze data.

## Results

A total of 164 ICU patients with septic shock were included; their characteristics are given in Table 1. Of the 164 included, 24 had died and 45 had shock resolution in the first 2 days after inclusion. Thus, 95 patients still had shock on the third day after inclusion. There were no differences between these 95 patients in admission SAPS II (54, IQR 48 to 63 versus 52, IQR 42 to 67, *P *= 0.54), SOFA score on day 1 (11, IQR 9 to 13 versus 10, IQR 8 to 13, *P *= 0.30), and 90-day mortality (52% versus 49%, *P *= 0.77) and the 69 patients who died or had shock resolution in the first 2 days after inclusion.

**Table 1:** Characteristics and selected therapies during shock in 164 consecutive intensive care unit patients with septic shock

Characteristics	*n *= 164
Males, number (percentage)	93 (57)
Age in years, median (IQR)	66 (59-74)
SAPS II, median (IQR)	54 (46-67)
Emergency surgery, number (percentage)	61 (37)
Estimated weight in kilograms on admission, median (IQR)	78 (65-93)
Focus of infection, number (percentage)	
Pulmonary	66 (40)
Gastrointestinal	46 (28)
Soft tissue	15 (9)
Urinary tract	9 (5)
Intravenous catheter	4 (2)
Other	5 (3)
Unclear	19 (12)
Shock duration in days^a^, median (IQR)	3 (2-5)
Therapies during septic shock, number (percentage)	
Invasive ventilation	147 (90)
Renal replacement therapy	65 (40)
Corticosteroids	79 (48)
Mortality, number (percentage)	
Day 30	65 (40)
Day 90	83 (51)

^a^The total shock duration in the intensive care unit. IQR, interquartile range; SAPS II, simplified acute physiology score II.

### Monitoring characteristics

All patients but one had plasma lactate measured on the first day of shock (median of 8 assessments per patient, IQR 6 to 10), and all patients in shock on the third day had lactate measured on day 3 (8 assessments per patient, IQR 6 to 9). One hundred twenty-five patients had ScvO_2 _measured on the first day of shock (2 assessments per patient, IQR 1 to 3), and 65 patients of the 95 still in shock on day 3 had ScvO_2 _measured (1 assessment per patient, IQR 1 to 2). No patients had a catheter for continuous ScvO_2 _measurements. On the first day of shock, 16 of the 164 patients had cardiac output measured; on the third day of shock, 19 of the 95 patients had cardiac output measured.

### Fluids given in the ICU on the first day of shock

The 164 included patients received a median of 4.0 L (IQR 2.3 to 6.3) of IV fluid in the ICU during the first day of septic shock. The patients receiving higher volumes of fluid (> 4.0 L) received more crystalloids, colloids, and blood products (Tables 2 and 3) compared with those receiving lower fluid volumes. The higher-volume group had a higher maximum value of plasma lactate and a tendency to higher maximum vasopressor dose compared with the lower-volume group (Table 2). In contrast, there were no differences between the two fluid volume groups in admission SAPS II, SOFA score on day 1, minimum ScvO_2_, and 90-day mortality (Table 2). Comparable results were obtained if fluid given as nutrition was added to the fluid volumes (data not shown).

**Table 2:** Characteristics dependent on fluid volume in the ICU in the first day and the first 3 days of shock in consecutive ICU patients with septic shock

	Day 1, *n *= 164			Day 1-3, *n *= 95		
	High fluid volume (> 4.0 L), *n *= 82	Low fluid volume (< 4.0 L), *n *= 82	*P *value	High fluid volume (> 7.5 L), *n *= 47	Low fluid volume (< 7.5 L), *n *= 48	*P *value
Total fluid volume, liters	6.3 (5.1-8.2)	2.3 (1.1-3.0)	< 0.0001	10.9 (8.7-13.3)	4.3 (3.0-5.7)	< 0.0001
Crystalloids, liters	3.9 (3.0-6.0)	2.0 (1.0-2.3), *n *= 59	< 0.0001	7.0 (4.5-9.8)	2.8 (0.5-4.3), *n *= 40	< 0.0001
Colloids, liters	1.1 (0.6-1.6), *n *= 72	0.5 (0.4-1.0), *n *= 64	< 0.0001	2.1 (1.0-2.5), *n *= 46	1.0 (0.5-1.5), *n *= 40	< 0.0001
Blood products, liters	1.2 (0.6-2.8), *n *= 53	0.5 (0.2-0.9), *n *= 34	< 0.0001	2.6 (1.1-4.3), *n *= 41	1.1 (0.5-1.9), *n *= 32	< 0.001
SAPS II on admission	54 (45-64)	54 (45-67)	0.73	53 (46-67)	55 (49-62)	0.47
SOFA score^a^	11 (9-13)	11 (9-13)	0.99	10 (8-13)	11 (10-14)	0.33
Maximum p-lactate in mmol/L^a^	3.4 (2.2-5.5)	2.0 (1.6-3.2), *n *= 81	< 0.0001	2.6 (1.7-3.4)	1.9 (1.6-2.4)	< 0.01
Minimum ScvO_2_, percentage^a^	70 (63-77), *n *= 68	73 (66-78), *n *= 57	0.26	74 (66-80), *n *= 34	74 (68-79), *n *= 31	0.35
Maximum vasopressor in μg/kg per minute^a^	0.25 (0.12-0.43)	0.18 (0.10-0.32)	0.07	0.16 (0.10-0.24)	0.15 (0.08-0.22)	0.57
Renal replacement therapy, number (percentage)	32 (39)	32 (39)	1.00	18 (38)	16 (33)	0.61
90-day mortality, number (percentage)	38 (46)	45 (55)	0.27	19 (40)	29 (62)	0.03

Values are presented as median (interquartile range) or as number of patients (percentage). Group comparisons were done by Mann-Whitney *U *test including all patients in the groups (82 versus 82 and 47 versus 48 patients) or chi-squared test by relevance. The number (n) is given in the specific cell that applies; for all other variables, n was as given in the top of the column. ^a^On days 1 and 3, respectively. ICU, intensive care unit; SAPS II, simplified acute physiology score II, ScvO_2_, central venous oxygen saturation, SOFA, sequential organ failure assessment.

**Table 3:** Details on fluids dependent on total fluid volume in the first day in the ICU and the first 3 days of shock in consecutive ICU patients with septic shock

	Day 1, *n *= 164			Day 1-3, *n *= 95		
Crystalloids	High fluid volume (> 4.0 L, *n *= 82)	Low fluid volume (< 4.0 L, *n *= 82)	*P *value	High fluid volume (> 7.5 L, *n *= 47)	Low fluid volume (< 7.5 L, *n *= 48)	*P *value
0.9% NaCl, liters	2.3 (1.0-4.0), *n *= 60	1.0 (0.6-1.6), *n *= 28	< 0.0001	3.0 (1.1-6.0), *n *= 35	1.0 (0.9-2.0), *n *= 19	< 0.01
Ringer solutions, liters	2.5 (2.0-4.0), *n *= 49	1.5 (1.0-2.0), *n *= 31	< 0.0001	3.1 (2.0-5.6), *n *= 34	2.0 (1.0-3.0), *n *= 29	< 0.01
Other electrolyte solutions, liters	1.0 (0.5-1.4), *n *= 32	1.0 (0.6-1.0), *n *= 22	0.48	1.5 (0.9-1.9), *n *= 29	1.0 (0.6-1.2), *n *= 18	0.14
Isotonic glucose, liters	1.0 (0.5-1.0), *n *= 16	0.5 (0.5-0.5), *n *= 4	0.02	1.0 (0.6-2.7), *n *= 14	0.5 (0.1-1.0), *n *= 3	0.10
Colloids, liters						
5% albumin	0.5 (0.4-1.0), *n *= 22	0.5 (0.3-0.8), *n *= 26	0.72	0.8 (0.5-1.5), *n *= 24	0.5 (0.4-1.0), *n *= 21	0.25
20% albumin	0.2 (0.1-0.3), *n *= 23	0.2 (0.2-0.3), *n *= 21	0.78	0.3 (0.1-0.4), *n *= 19	0.2 (0.2-0.4), *n *= 20	0.53
6% HES 130/0.4	1.0 (0.5-1.5), *n *= 52	0.5 (0.5-1.0), *n *= 23	< 0.0001	1.5 (1.0-2.0), *n *= 35	1.0 (0.5-1.5), *n *= 14	< 0.0001
6% dextran 70	1.0 (0.9-1.0), *n *= 10	0.8 (0.5-1.0), *n *= 9	0.76	1.0 (0.5-1.2), *n *= 12	0.6 (0.5-1.0), *n *= 12	0.90
Blood products, liters						
Red blood cells	0.7 (0.5-1.2), *n *= 48	0.3 (0.2-0.5), *n *= 22	< 0.0001	1.0 (0.6-1.8), *n *= 39	0.5 (0.3-0.8), *n *= 20	< 0.0001
Fresh frozen plasma	0.6 (0.4-1.3), *n *= 38	0.5 (0.5-0.6), *n *= 13	< 0.0001	1.4 (0.9-1.8), *n *= 33	1.1 (0.5-1.1), *n *= 17	< 0.0001
Platelet concentrate	0.7 (0.4-1.3), *n *= 17	0.7 (0.4-1.1), *n *= 7	0.03	0.7 (0.6-1.4), *n *= 20	1.2 (0.7-1.6), *n *= 8	0.02^a^

Values are presented as median (interquartile range) of the number given in the specific cell; this number denotes the number of patients given the specific solution. Group comparisons were done by Mann-Whitney *U *test including all patients in the groups (82 versus 82 and 47 versus 48 patients). ^a^The higher-volume group received significantly more platelets (*P *= 0.02) because more patients received platelets compared with the lower-volume group (20 versus 8 patients). HES, hydroxyethyl starch (molecular weight/substitution degree).

### Nutrition given in the ICU on the first day of shock

A median of 0.5 L (IQR 0.2 to 1.1) of nutrition was given on the first day of shock, and the higher-fluid volume group received less nutritional volume than the lower-fluid group (0.4 L, IQR 0.1 to 0.9 versus 0.8 L, IQR 0.2 to 1.3, *P *= 0.0009).

### Cumulative urinary output and fluid balance in the ICU on the first day of shock

The cumulative urinary output was 1.3 L (IQR 0.4 to 2.4) by the end of the first day of shock without a difference between the higher- and lower-fluid volume groups (1.2 L, IQR 0.4 to 2.1 versus 1.7 L, IQR 0.4 to 2.6, *P *= 0.46). The cumulative fluid balance was 3.4 L (IQR 1.5 to 5.6) by the end of the first day. The patients receiving higher volumes of fluid had a higher cumulative fluid balance in the first day of shock compared with those receiving lower fluid volumes (5.6 L, IQR 4.1 to 7.3 versus 1.9 L, IQR 0.5 to 2.9, *P *< 0.0001).

### Fluids given in the ICU in the first 3 days of shock

The 95 patients who still had shock on the third day after inclusion had received a median of 7.5 L (IQR 4.3 to 10.8) of IV fluid by the end of the third shock day. The patients receiving higher fluid volumes (> 7.5 L) in the first 3 days of shock received more crystalloids, colloids, and blood products compared with those receiving lower fluid volumes and had a higher maximum plasma lactate on day 3 (Tables 2 and 3). The two groups had comparable admission SAPS II and SOFA scores, maximum vasopressor dose, and minimum ScvO_2 _on day 3 (Table 2), but the higher-fluid volume group had a lower 90-day mortality than the lower-fluid volume group (Table 2). Comparable results were obtained if fluid given as nutrition was added to the fluid volumes (data not shown).

### Nutrition given in the ICU in the first 3 days of shock

A median of 3.2 L (IQR 2.3 to 4.2) of nutrition was given on the first 3 days of shock, and the higher-fluid volume group received a significantly lower volume of nutrition compared with the lower-fluid volume group (2.9 L, IQR 2.1 to 3.9 versus 3.4 L, IQR 2.6 to 4.4, *P *= 0.04).

### Cumulative urinary output and fluid balance in the ICU in the first 3 days of shock

In the 95 patients who still had shock on the third day, the cumulative urinary output was 5.7 L (IQR 1.8 to 8.7) by the end of that third day without a difference between the higher- and lower-fluid volume groups (5.6 L, IQR 1.9 to 8.7 versus 5.9 L, IQR 0.7 to 9.1, *P *= 0.67). The cumulative fluid balance was 5.4 L (IQR 2.7 to 9.4) by the end of that third day. The patients receiving higher volumes of fluid had a higher cumulative fluid balance in the first 3 days of shock compared with those receiving lower fluid volumes (9.2 L, IQR 5.3 to 13.6 versus 2.9 L, IQR 0.9 to 5.4, *P *< 0.0001).

## Discussion

The main finding of this study was that, in comparison with patients receiving lower volumes of fluid, those receiving higher initial fluid volumes had higher lactate levels but the same 90-day mortality. In contrast, the combination of higher volumes of crystalloids, colloids, and blood products was associated with lower mortality in the subgroup of patients with shock lasting 3 days or more. This was surprising because analyses from the SOAP and VASST (Vasopressin and Septic Shock Trial) cohorts have shown the opposite result.

In the SOAP study [4], positive fluid balance on the third day after the onset of infection was associated with increased ICU mortality in the subgroup of patient with sepsis, among whom 40% had septic shock. Recent results from VASST [5] also indicated that a positive fluid balance at both 12 hours and day 4 was associated with increased 28-day mortality. The VASST cohort was highly selected as only 13% of screened patients were included. In neither of these two studies was there information on the types of fluid given. Also, multivariate analyses were used to show the effect of fluid balance on outcome in the VASST and SOAP cohorts. Such analyses may be difficult to construct for complex interventions in complex settings. The challenges are confounding by indication, time dependency, and repeated exposure of the intervention and competing risks. In contrast, we used unadjusted analyses in the present study. Some of the above potential biases also hamper the interpretation of our results. In particular, the day 3 cohort may be subjected to bias because these patients survived to day 3 but still had shock. The systematic exclusion of the most sick (those who died) and the least sick (those who had shock resolution) may have introduced selection biases, which are difficult to assess. On the other hand, the persisting shock group had admission SAPS II, SOFA score on day 1, and 90-day mortality that were comparable to those of the group of dropouts.

Also, a retrospective cohort study by Murphy and colleagues [6] assessed potential effects of fluid volumes. The study was of patients with septic shock, but again the cohort was selected, as acute lung injury was an inclusion criterion and there were several exclusion criteria. This study indicated that higher initial IV fluid volumes were associated with lower mortality if negative fluid balance was obtained on two consecutive days within the first 7 days of shock [6].

The findings in the present study are partly supported by those of studies of fluid resuscitation in the first 6 hours of patients with severe sepsis in Canadian ICUs [12] and in the first 24 hours of patients with septic shock in Scandinavian ICUs [8]. Neither of these studies found outcome to be associated with initial volumes of IV fluids.

Direct comparison between the previous studies and our study is difficult, because we focused on IV fluid volume given to unselected patients with septic shock, allowing detailed analyses of those with persistent shock. Also, we divided patients into groups receiving higher and lower volumes of fluids. Hereby, we observed that patients receiving higher fluid volumes had higher lactate levels, indicating hypoperfusion and the need for intervention. This may explain the potential benefit of fluid in the higher-volume group with persisting septic shock. In any case, clinicians should interpret results of cohort studies with caution before making inferences on treatment. But until we have data from RCTs on fluid volume in septic shock, patients with persisting shock and hypoperfusion should be carefully assessed for hypovolemia and fluid challenged as recommended in the SSC guidelines [2].

### Colloid use

The composition of resuscitation fluids for sepsis is controversial. In particular, the use of colloid is a matter of debate [13]. In the present study, the administration of colloids (in particular, HES 130/0.4) was not associated with increased mortality or use of dialysis. This contrasts with results from a recent before-and-after study in patients with severe sepsis in a single ICU [13], where use of HES 130/0.4 was associated with kidney injury and a tendency to increased use of dialysis. However, the doses of HES 130/0.4 appeared to be higher in that study (median of 46 mL/kg during ICU stay) compared with ours (13 mL/kg in the first day of shock and 19 mL/kg for those who still had shock on day 3). Neither of the two large RCTs comparing colloids with crystalloids showed significant changes in mortality [14,15]. These divergent results underline the need for large RCTs on the efficacy and safety of colloid treatment in patients with sepsis, in particular 6% HES 130/0.4, as this solution is largely unstudied in these patients. Two large RCTs randomly assigning hypovolemic ICU patients to 6% HES 130/0.4 versus crystalloids are ongoing [16,17].

### Blood product use

The use of blood products in the resuscitation of patients with sepsis is controversial, but in the present study these were not associated with worse outcome. In contrast, the patients who had persisting septic shock and who also received higher volumes of RBCs, plasma, and platelets had better survival. There are hardly any data on the efficacy and safety of plasma and platelet therapy in patients with sepsis, but the two trials randomly assigning patients with sepsis to different RBC transfusion strategies have shown divergent results. Rivers and colleagues [3] found increased survival with a complex early goal-directed protocol including RBC transfusion if hypoperfusion persisted. On the other hand, the TRICC (Transfusion Requirements in Critical Care) trial found a tendency toward increased mortality with liberal RBC transfusion in the subgroup of patients with sepsis [18], but these were included only after resuscitation. Non-leukocyte-depleted RBCs were used in the TRICC trial, whereas Danish hospitals use pre-storage leukocyte-depleted RBCs suspended in saline-adenine-glucose-mannitol (SAGM). Again, large RCTs are needed to test the efficacy and safety of RBCs, fresh frozen plasma, and platelet concentrates in patients with sepsis.

### Strengths and weaknesses

The strengths of this study include prospective inclusion of consecutive patients at multiple university and non-university ICUs using a well-defined definition of septic shock, detailed registration of fluids given, and follow-up using national identification numbers, which ensured close to full follow-up.

There are clear limitations to this study. First of all, the descriptive design precludes strong conclusions regarding effects of fluid volume in patients with sepsis. Also, data on co-morbidities, pre-ICU interventions, some co-interventions, and subsequent interventions after the shock period were not registered. The specific timing and rate of fluid infusion were not assessed, nor were the specific therapy triggers and goals that clinicians used. Also, we did not register if central venous pressure was assessed and registered, because we had previously shown that this marker of preload is used less during resuscitation of patients with septic shock in Danish ICUs [8]. On the other hand, the evidence to support the use of any specific trigger and goal for fluid resuscitation of ICU patients with septic shock is weak, in particular beyond the first 6 hours [2].

## Conclusions

In this cohort of unselected patients with septic shock, initial fluid volume was not associated with mortality. In patients with shock for 3 days or more, higher fluid volumes including crystalloids, colloids, and blood products were associated with reduced mortality. This contrasts with results from other cohort studies, underlining the need for RCTs on fluid volume in septic shock.

## Key messages

• Septic shock patients who received higher intravenous (IV) fluid volumes, including crystalloids, colloids, and blood products, on day 1 had higher p-lactate on day 1.

• Mortality was independent of IV fluid volume on day 1 of septic shock.

• Persisting-shock patients who received higher cumulated IV fluid volumes, including crystalloids, colloids, and blood products, at the end of day 3 had higher p-lactate on day 3.

• In patients with persisting shock, mortality was lower in the group receiving higher IV fluid volume than in those receiving lower fluid volume.

## Abbreviations

HES: hydroxyethyl starch; ICU: intensive care unit; IQR: interquartile range; IV: intravenous; RBC: red blood cell; RCT: randomized clinical trial; SAPS II: simplified acute physiology score II; ScvO_2_: central venous oxygen saturation; SOAP: Sepsis Occurrence in Acutely Ill Patients; SOFA: sequential organ failure assessment; SSC: Surviving Sepsis Campaign; TRICC: Transfusion Requirements in Critical Care; VASST: Vasopressin and Septic Shock Trial.

## Competing interests

AP receives research support from B Braun Medical (Bethlehem, PA, USA) and Fresenius Kabi (Bad Homburg, Germany) and honoraria for being part of the steering committee of a sepsis trial by Ferring Pharmaceuticals (Copenhagen, Denmark). SHS declares that he has no competing interests.

## Authors' contributions

Both authors contributed to study design, data analyses, and revision of the manuscript. SHS collected the data and established the database. AP drafted the first version of the manuscript. Both authors read and approved the final manuscript.
